# Development of a rapid lateral flow assay for detection of anti-coccidioidal antibodies

**DOI:** 10.1128/jcm.00631-23

**Published:** 2023-09-01

**Authors:** Francisca J. Grill, Sergei Svarovsky, Maria Gonzalez-Moa, Erin Kaleta, Janis E. Blair, Lydia Lovato, Richard Grant, Kyle Ross, Barbara K. Linnehan, Jenny Meegan, Kenta S. Reilly, Ashlyn Brown, Stacy Williams, Yunro Chung, D. Mitchell Magee, Thomas E. Grys, Douglas F. Lake

**Affiliations:** 1 School of Life Sciences, Arizona State University, Tempe, Arizona, USA; 2 Glycodots LLC, San Diego, California, USA; 3 Department of Laboratory Medicine and Pathology, Mayo Clinic, Phoenix, Arizona, USA; 4 Division of Infectious Diseases, Mayo Clinic, Phoenix, Arizona, USA; 5 Veterinary Neurological Center, Phoenix, Arizona, USA; 6 Washington National Primate Research Center, Seattle, Washington, USA; 7 National Marine Mammal Foundation, San Diego, California, USA; 8 Center for Personalized Diagnostics, Biodesign Institute, Arizona State University, Tempe, Arizona, USA; 9 College of Health Solutions, Arizona State University, Phoenix, Arizona, USA; University of Utah, Salt Lake City, Utah, USA

**Keywords:** Valley fever, coccidioidomycosis, diagnostic, LFA

## Abstract

*Coccidioides* spp. are dimorphic fungi that are capable of infecting human and non-human mammals and can cause diverse manifestations of coccidioidomycosis or Valley fever (VF). In combination with clinical symptoms and radiographic findings, antibody-based diagnostic tests are often used to diagnose and monitor patients with VF. Chitinase 1 (CTS1) has previously been identified as the seroreactive antigen used in these diagnostic assays to detect anticoccidial IgG. Here, an indirect enzyme-linked immunosorbent assay to detect IgG to CTS1 demonstrated 165 of 178 (92.7%) patients with a positive result by immunodiffusion (ID) and/or complement fixation (CF) had antibodies to the single antigen CTS1. We then developed a rapid antibody lateral flow assay (LFA) to detect anti-CTS1 antibodies. Out of 143 samples tested, the LFA showed 92.9% positive percent agreement [95% confidence interval (CI), 84.3%–96.9%] and 97.7% negative percent agreement (95% CI, 87.9%–99.6%) with ID and CF assays. Serum or plasma from canines, macaques, and dolphins was also tested by the CTS1 LFA. Test line densities of the CTS1 LFA correlated in a linear manner with the reported CF and ID titers for human and non-human samples, respectively. This 10-min point-of-care test for the rapid detection of anti-coccidioidal antibodies could help to inform healthcare providers in real-time, potentially improving the efficiency of healthcare delivery.

## INTRODUCTION

Coccidioidomycosis, or Valley fever (VF), is a fungal infection caused by *Coccidioides* species that is primarily endemic to southern Arizona and the San Joaquin Valley region of California, but also occurs in arid regions of the western USA, Central America, and South America (1, 2). Humans and other vertebrates are susceptible to infection with *Coccidioides* through the inhalation of airborne arthroconidia into the lungs (3, 4). Other non-human animals reported to be infected with *Coccidioides* include, but are not limited to, primates (5 - 9), canines (10 - 12), and marine mammals under human care such as dolphins (13). About two-thirds of human and canine VF cases are subclinical (14 - 16). The remainder of infections typically manifests as a community-acquired pneumonia (CAP) with nonspecific symptoms and radiographic imaging. Coccidioidomycosis has been implicated to cause 15%–30% of CAP infections in endemic regions, though the proportion of CAP patients tested for VF is reportedly low (17, 18). Consequently, unnecessary antibacterial medications are often prescribed prior to a VF diagnosis being made (17, 19).

Diagnosis of VF infection is often achieved through serologic antibody testing in the clinical laboratory accompanied by clinical and radiological findings (20, 21). The routine methods used for the evaluation of patients with suspect VF are enzyme immunoassay (EIA), immunodiffusion (ID) assay, and complement fixation (CF) assay; all three methods detect anti-coccidioidal antibodies. Both EIA and ID employ reagents that may distinguish immunoglobulin (Ig) type, with IgM typically present early in infection, followed later by an IgG response (22, 23). Qualitative detection of anti-*Coccidioides* IgM and IgG can be performed in 2–3 hours by EIA, which is utilized in many clinical laboratories. While the value of detecting IgM and/or IgG by EIA has been shown, others have reported a markedly high false-positive rate of EIA IgM detection (24 - 26). Compared to EIA, ID is generally more specific while CF can sometimes be more sensitive, but both have a slower turnaround time (24–48 hours) and require materials and expertise that are generally found only in complex or reference testing laboratories (27). These analytical assays may be performed only after pre-analytic steps such as specimen transportation and processing (centrifugation, separation, and data entry). Post-analytical steps (data entry, physician review, and patient communication) add additional time to the test process, extending the time until therapeutic decisions can be made (28).

Antigens utilized in commercial *Coccidioides* diagnostic assays are proprietary. However, antigen preparations are generally thought to be composed of multiple antigens from *Coccidioides* species cultures. Chitinase 1 (CTS1) was previously identified as a seroreactive component in CF antigen preparations (29 - 31), the reagent used to detect anti-coccidioidal IgG. The seroreactive antigen in tube-precipitin antigen preparations used to detect anti-coccidioidal IgM is less characterized, though IgM reactivity has been identified against β-glucosidase 2, a glycoprotein with 3-*O*-methylmannose glycosylations that appear essential for seroreactivity of patient IgM antibodies (32). Different antigen preparations and different methods of each serodiagnostic assay likely explain the variable performances of EIA, ID, and CF, with no single assay consistently superior, often resulting in their combined use to aid in VF diagnosis. Still, ID and CF can provide additional value to testing strategies, since both can estimate an antibody titer that is often used with clinical signs in the evaluation and management of patients with VF (33).

An ideal VF diagnostic test would be a sensitive and specific antigen-based assay to directly detect the *Coccidioides* fungus. To date, there are only two assays described that detect coccidioidal antigen in routine clinical specimens (e.g., serum and urine). One assay measures serum 1,3-β-d-glucan (BDG) levels, and the other measures circulating *Coccidioides* galactomannan in serum or urine (34, 35). BDG is also detected in patients with histoplasmosis, blastomycosis, and aspergillosis and is therefore not specific to *Coccidioides*, while the test for *Coccidioides* galactomannan has also been shown to cross-react (34 - 37). Thus, in the absence of an accurate antigen test for VF, one way to increase value of current antibody tests would be improvement in their sensitivity and speed to allow for appropriate care of VF patients and a reduction in unnecessary antibiotic use. Currently, there is one lateral flow assay (LFA) commercially available to aid in the diagnosis of VF called sōna (IMMY, Norman, OK, USA). The sōna LFA can return a result within 1 hour and has been reported to have a sensitivity of 31%–93% when compared to EIA (38, 39). The result of the sōna LFA is interpreted visually, introducing subjectivity to the result. Nonetheless, this LFA has shown potential value in using a rapid test in clinical settings to allow for earlier VF diagnosis and subsequently aid in reducing unnecessary antibiotic use (38). Additionally, two studies with canine sera have shown the sōna LFA to have moderate to good agreement with ID, the current serologic reference standard in dogs (40, 41). Upon review of the literature, sōna has not been evaluated in other non-human animals; however, a rapid LFA that can return a result comparable to ID in a fraction of the time could be valuable in veterinary settings.

In an effort to improve VF diagnostic speed while maintaining accuracy, we present the development and evaluation of a semi-quantitative host species-agnostic LFA which takes 10 minutes to obtain a result and can currently accommodate both serum and plasma. We evaluated the test using 143 human, 50 canine, 33 macaque, and 15 dolphin serum or plasma samples. Results from the LFA were compared to clinical reference methods.

## MATERIALS AND METHODS

### Human specimens

Human serum samples from an endemic area (*n* = 294, Phoenix, AZ, USA) were tested by three *Coccidioides* antibody assays (EIA, ID, and CF) as part of routine patient care. Serum samples were collected between February and October 2022 and stored at −20°C or −80°C until use. These 294 sera were collected as an untargeted convenience sample from routine testing and include the following: (i) 116 sera from individuals who were positive for IgG and/or IgM by EIA, ID, and CF; (ii) 62 sera from individuals who were IgG and/or IgM positive by two of these methods (i.e., EIA/ID, EIA/CF, and ID/CF); (iii) 30 sera from individuals who were IgG positive by EIA only; and (iv) 86 sera from individuals who were negative for IgG and IgM by all methods. An additional 29 samples were provided as nonendemic and other mycoses controls by Dr. Elitza Theel (Mayo Clinic, Rochester, MN, USA). These 29 sera include the following: 11 sera from patients positive for *Aspergillus* galactomannan antigen, three sera from patients with positive serology by *Blastomyces* EIA and ID assays, 10 sera from patients with positive serology by *Histoplasma* ID, and five sera from apparently healthy individuals. All 323 human sera were tested by indirect enzyme-linked immunosorbent assay (ELISA) for anti-CTS1 IgG antibodies as detailed below. Of the 323 sera tested by the CTS1 ELISA, 143 were randomly selected to be tested by the CTS1 LFA described below. Human sera were collected under a Mayo Clinic institutional review board (IRB)-approved protocol no. 12-000965.

### Detection of CTS1 IgG antibodies by ELISA

A total of 323 human serum samples were tested for the presence of CTS1 IgG antibodies by an ELISA performed in our research laboratory. Recombinant CTS1 (rCTS1) was coated on ELISA plates at 2 µg/mL overnight at 4°C. The next day, plates were allowed to equilibrate to room temperature for 10 minutes followed by three washes with 1× phosphate-buffered saline + 0.05% Tween-20 (PBST). Plates were blocked with 1% bovine serum albumin (BSA) for 1 hour. Human sera were diluted 1:100 in 1% BSA, and a previously published humanized anti-CTS1 monoclonal antibody (4H2) was used as a positive control at 1 µg/mL (42, 43). All samples were run in duplicate. After 1-hour incubation with diluted sera, plates were washed three times with PBST followed by addition of horseradish peroxidase-conjugated goat anti-human IgG Fc-specific antibody (1:10,000 dilution, Sigma-Aldrich). Plates were washed four times with PBST and then developed with 3,3’,5,5’-tetramethylbenzidine (TMB). The reaction was stopped with 0.16 M sulfuric acid and absorbance was read at 450 nm. The cutoff was determined by calculating the mean and standard deviation (SD) optical density (OD_450_) values of all samples reported negative by EIA, ID, and CF as follows: Cutoff = mean_neg_ + 2SD_neg_.

### Nonhuman specimens

Canine serum samples consisted of 29 samples from dogs residing in the endemic area (provided by LL) and 21 samples from dogs residing in the Tidewater region of eastern Virginia (purchased from North American Veterinary Blood Bank, Manassas, VA, USA). Endemic canine sera were drawn as part of an initial patient evaluation, banked at the time of their collection, and were not specifically collected for this study. Endemic canine samples were chosen if they had a prior IgG ID titer of 1:8 or greater (reported by various laboratories) and/or exhibited neurological signs (seizures or paresis suggesting central nervous system involvement) or magnetic resonance imaging findings consistent with coccidioidal granuloma (44). Pig-tailed macaque (*Macaca nemestrina*) plasma samples (*n* = 33) were provided by RG and included animals within the endemic area with a positive antibody titer by *Coccidioides* ID (Protatek Reference Laboratory), animals within the endemic area with a past positive antibody titer by ID that had regressed, and animals born and raised in a nonendemic area (Seattle, WA, USA) with no exposure to the endemic region. Macaque samples had been heated to 56°C for 30 minutes and were shipped frozen on dry ice. Bottlenose dolphin (*Tursiops truncatus*) serum samples (*n* = 15) were provided by KR, BL, and JM. All samples were collected during routine clinical care as part of the preventative medicine program for animals at the US Navy Marine Mammal Program in San Diego, CA, and banked at the time of their collection. Dolphin samples were then selected as follows: five samples from a dolphin with coccidioidomycosis, four of which produced a positive antibody titer by *Coccidioides* ID (University of California Davis); five samples from dolphins with various fungal diseases (aspergillosis, cryptococcosis, mucormycosis, and candidiasis); and five samples from healthy dolphins without known fungal disease based on physical exams, quarterly bloodwork, and a comprehensive record review in the year preceding and following the date of the provided sample. All dolphins with fungal disease were diagnosed by culture or PCR. Neither macaque nor dolphin samples were collected specifically for this study. Both macaque and dolphin samples were de-identified and run in a blinded manner. After macaque and dolphin results were reported to RG and KR, respectively, sample information was revealed.

### *Coccidioides* immunodiffusion

The canine, macaque, and dolphin samples used in this study came from various institutions that utilize different diagnostic laboratories for ID. Therefore, ID and quantitative immunodiffusion (QID) were performed for all samples using the same reagents. The ID and QID assays had previously been compared to commercial assays employed at a reference laboratory (Mayo Clinic Laboratories) and shown to be equivalent (data not shown). The initial screening of samples by ID was set up using a standard protocol. Briefly, 20 μL of positive control goat antisera was added to the top and bottom wells, then 20 μL of non-human patient plasma or sera was added to the remaining four outer wells of the immunodiffusion plate. Then, 20 μL of *Coccidioides* antigen was added to the center well. The plates were incubated in a humidified chamber at 27°C for 48 hours and then read for the presence of a line of precipitin between the patient and antigen wells. The absence of a line or presence of a line that did not interact with the adjacent positive control goat antisera was recorded as negative. Any patient who had a positive result was subsequently run by QID, whereby serum or plasma was twofold serially diluted, run in the ID test as previously described, and antibody titer determined by the dilution at which the line of precipitin is last detected.

### CTS1 antibody LFA

**Fig 1:**
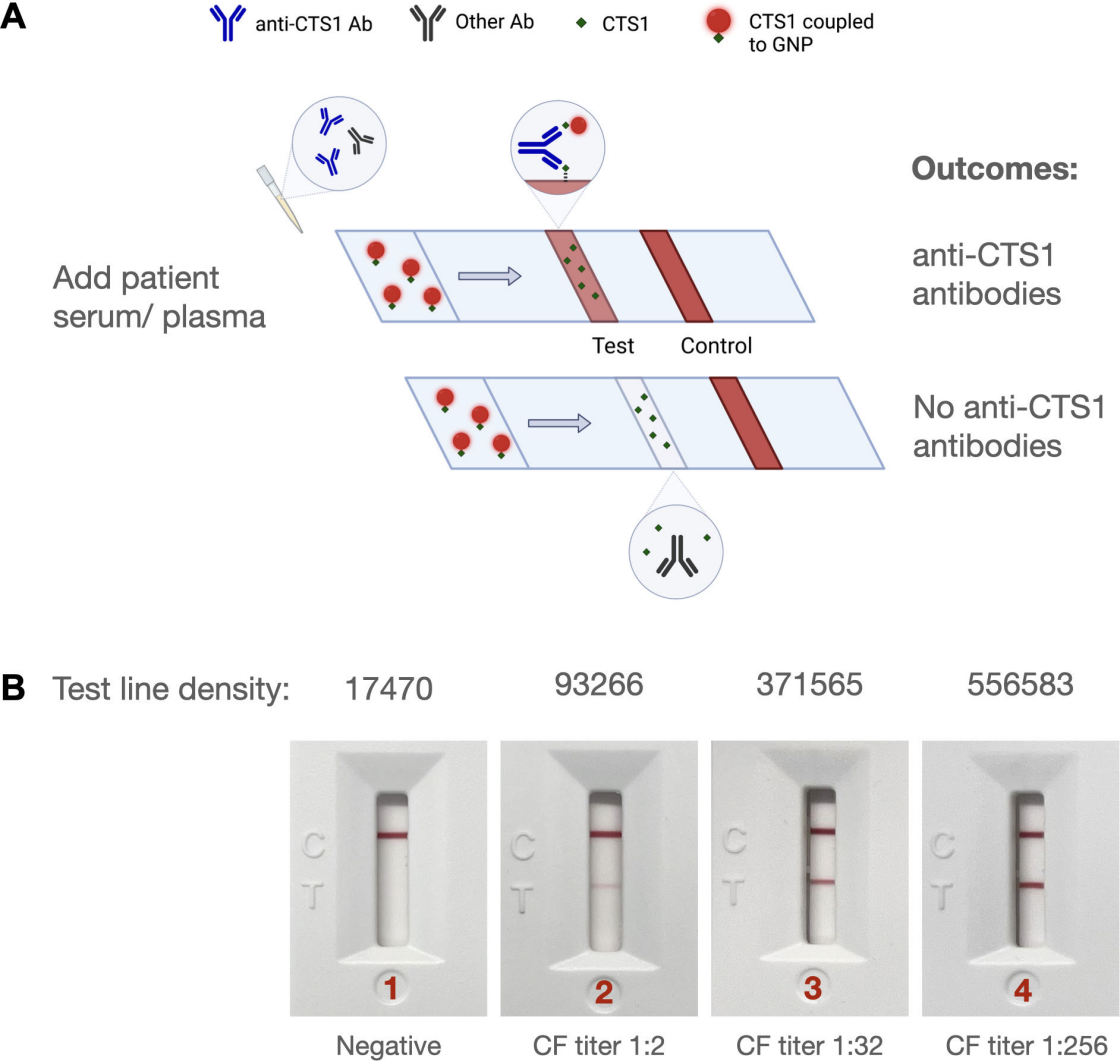
(A) Schematic of anti-CTS1 antibody LFA. Patient serum or plasma is added to the sample pad and chased with buffer to promote lateral flow. If anti-CTS1 antibodies are present, a mixture of antibody complexes to CTS1 and CTS1 coupled to gold nanoparticles (GNP) will be formed, creating a visible test line. If no anti-CTS1 antibodies are present, no complexes will be formed, and no test line will be observed. (B) Examples of LFA results for four patients. The red control line adjacent to the “C” on the cassette indicates the test ran properly, while the presence or absence of a red line adjacent to the “T” on the cassette is used to measure the level of anti-CTS1 antibodies in the sample tested. (1) represents a patient with a negative result with test line density units of 17,470; (2) represents patient serum with a test line density of 93,266 and CF titer of 1:2; (3) represents patient serum with a test line density of 371,565 and CF titer of 1:32; and (4) represents patient serum with a test line density of 5,56,583 and CF titer of 1:256. CF titers are those reported by the reference laboratory (Mayo Clinic Laboratories).

The CTS1 LFA was developed to detect and measure levels of antibodies in serum for patients with suspected VF. The LFA assay strip contains a sample pad, a conjugate pad, a nitrocellulose membrane striped with test and control lines, and an absorbent pad (GlycoDots, LLC). The assay is designed as a bridging lateral flow assay, whereby the bivalent nature of immunoglobulin is utilized. Briefly, serum is added to the sample port followed by addition of sample buffer that carries patient serum toward the test line. If anti-CTS1 antibodies are present, they will bridge CTS1 coupled to colored nanoparticles with CTS1 at the test line on the strip. The semi-quantitative nature of the test allows for increasing test line density as anti-CTS1 antibodies’ levels increase. A schematic of this assay design is shown in Fig. 1A.

To perform the CTS1 LFA, 6.8 µL of serum or plasma (the corresponding volume to 10 µL of whole blood) was added to the sample pad, followed by 60 µL of chase buffer. After 10 minutes, densities of both the test and control lines were read using an iDetekt RDS-2500 density reader (Detekt Biomedical, Austin, TX, USA). A red control line indicates the test ran appropriately (also reported as density units), while the density units of the test line may be used to approximate the levels of anti-CTS1 antibodies present in the sample being tested. The test is visually intuitive, whereby the absence of a test line is indicative of a negative result, and the presence of a test line indicates a positive result. The subjectivity of test line visualization for samples from patients with low antibody levels is removed through the density unit readout provided by the LFA reader. Precision testing was performed on a single lot of test strips using sera from one negative and one positive patient in replicates of 10 and calculating the coefficient of variation (CV) as a percentage as follows: %CV = (standard deviation/mean) × 100%.

### *Coccidioides* antibody LFA (sōna)

A commercially available *Coccidioides* antibody lateral flow assay (sōna, IMMY, Norman, OK, USA) was purchased and run according to manufacturer’s instructions. Briefly, sera were diluted 1:441 by first adding 10 µL of serum into 200 µL of the provided specimen diluent, then adding 10 µL of the first dilution to a second tube with 200 µL of specimen diluent. A 100 µL volume of each 1:441 dilution was transferred to a flat-bottom 96-well plate (Corning) and a sōna strip was inserted into each well. Tests were allowed to incubate for 30 minutes and were subsequently interpreted by two independent observers within 60 minutes of incubation. Each strip should produce a perceptible pink/red control line to be considered valid. If there is any perceptible second pink/red line in the test region of the strip, the test result is positive. The presence of only a control line, or a control line with a gray test line, is a negative result. If there was disagreement between the result reported by each independent observer, the test was re-run and read by three independent observers with the majority result (i.e., 2/3) reported as the result. Quality control of each sōna kit was performed by using three drops of the provided *Coccidioides* Ab positive control (positive result QC) or 100 µL of specimen diluent (negative result QC).

### Data analysis

For the CTS1 LFA, receiver operating characteristic (ROC) analysis was performed to determine the optimal test line density unit cutoff value. For each species, we compared test line density unit values across groups categorized by diagnostic results using the Kruskal-Wallis test and performed *post hoc* tests using Wilcoxon rank sum test with Bonferroni correction. Spearman’s correlation (*r_s_*) was conducted to analyze the association between CF titer and LFA density units (45). For the CTS1 and sōna LFAs, we calculated the positive percent agreement (PPA), negative percent agreement (NPA), positive predictive value (PPV), negative predictive value (NPV), and their respective 95% confidence intervals (CIs). False discovery rate-adjusted *P* values less than 0.05 were classified as statistically significant. We used GraphPad Prism 6.0 and R 4.2.1 statistical software for these analyses.

## RESULTS

### Establishment of CTS1 as a primary seroreactive protein

Since the use of CTS1 as a single antigen is not well characterized, we performed an ELISA using recombinant CTS1 and tested serum samples from patients with positive VF serology as well as negative control serum samples. Three hundred twenty-three human serum samples were tested in the CTS1-based ELISA to determine the prevalence of anti-CTS1 antibodies. The 323 samples were divided into three groups as follows: patients positive by CF and/or ID (*n* = 178); patients negative by CF and ID (*n* = 121) which was further split into patients who were IgG positive by EIA (30/121) and negative by EIA (91/121); and nonendemic samples from patients with positive serology to *Aspergillus*, *Blastomyces*, or *Histoplasma* serologic assays (*n* = 24). Using the OD_450_ values from patients negative by CF, ID, and EIA and those with other mycoses (*n* = 115), the cutoff for positivity for this CTS1 ELISA was calculated to be an OD_450_ of 0.44.

**Fig 2:**
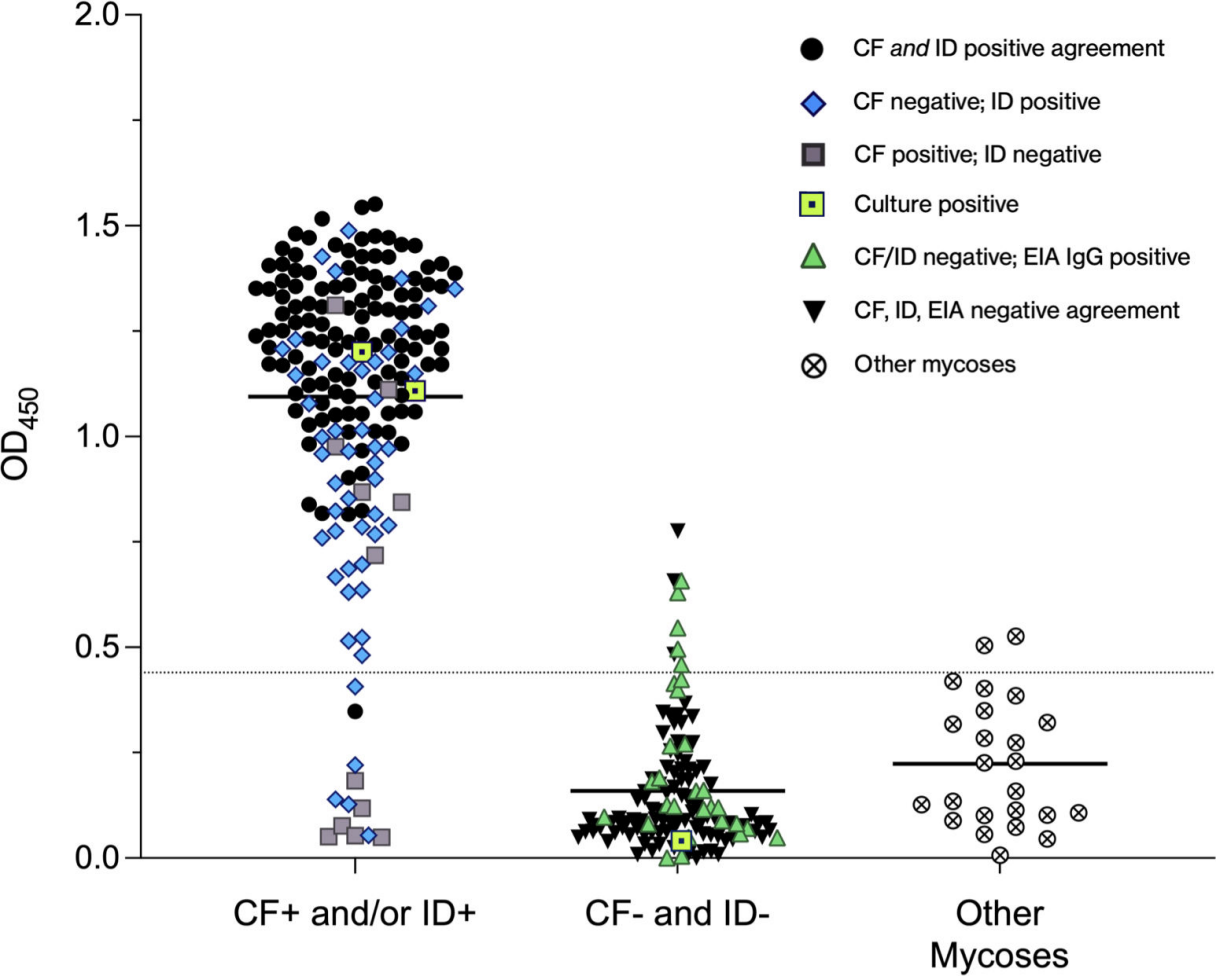
Prevalence of anti-CTS1 antibodies in human sera. The group on the left of the graph includes patients who were positive by CF and/or ID: samples that were positive by both CF and ID are represented by black circles; samples positive by CF but negative by ID (IgG and/or IgM) are shown as grey squares; and samples negative by CF but positive by ID (IgG and/or IgM) are shown as blue diamonds. The group in the middle of the graph includes patients who were negative by CF and ID: samples that were positive by EIA IgG are shown as green triangles and samples negative by EIA are shown as black inverted triangles. The group on the right of the graph shows patients with positive serology to other endemic mycoses represented with an X encompassed in a circle. Three patients tested were *Coccidioides* culture positive and are denoted as yellow squares with a black center. A legend with all symbols and their meaning is included in the upper right portion of the graph. The cutoff for positivity is designated on the graph as a dotted line.

Of 178 samples positive by CF and/or ID, 166 (93.3%) returned a positive result for anti-CTS1 antibodies and 116 of 117 (99.1%) positive by both CF and ID were positive (Fig. 2). Only 5 of 32 patients (15.6%) who were negative by CF and ID but positive by EIA had anti-CTS1 antibody levels considered positive in our CTS1 ELISA. Two of 24 patients (8.3%) with positive serology to other endemic mycoses returned a positive result slightly above the cutoff threshold.

### Performance of CTS1 antibody lateral flow assay

After determining that 99.1% of patients who were positive by both CF and ID (116/117) make antibodies to CTS1 detectable in our CTS1 ELISA, we sought to translate this assay into a rapid LFA. As shown in Fig. 1A, the configuration of the LFA is such that serum containing anti-CTS1 antibodies produces a red test line which can vary in intensity depending on the levels of anti-CTS1 antibodies (IgG or IgM) in serum or plasma. Figure 1B demonstrates the results of the LFA for serum from VF patients with different levels of CF/CTS1 antibodies, with the test line intensity increasing correlatively with CF titer.

**Fig 3:**
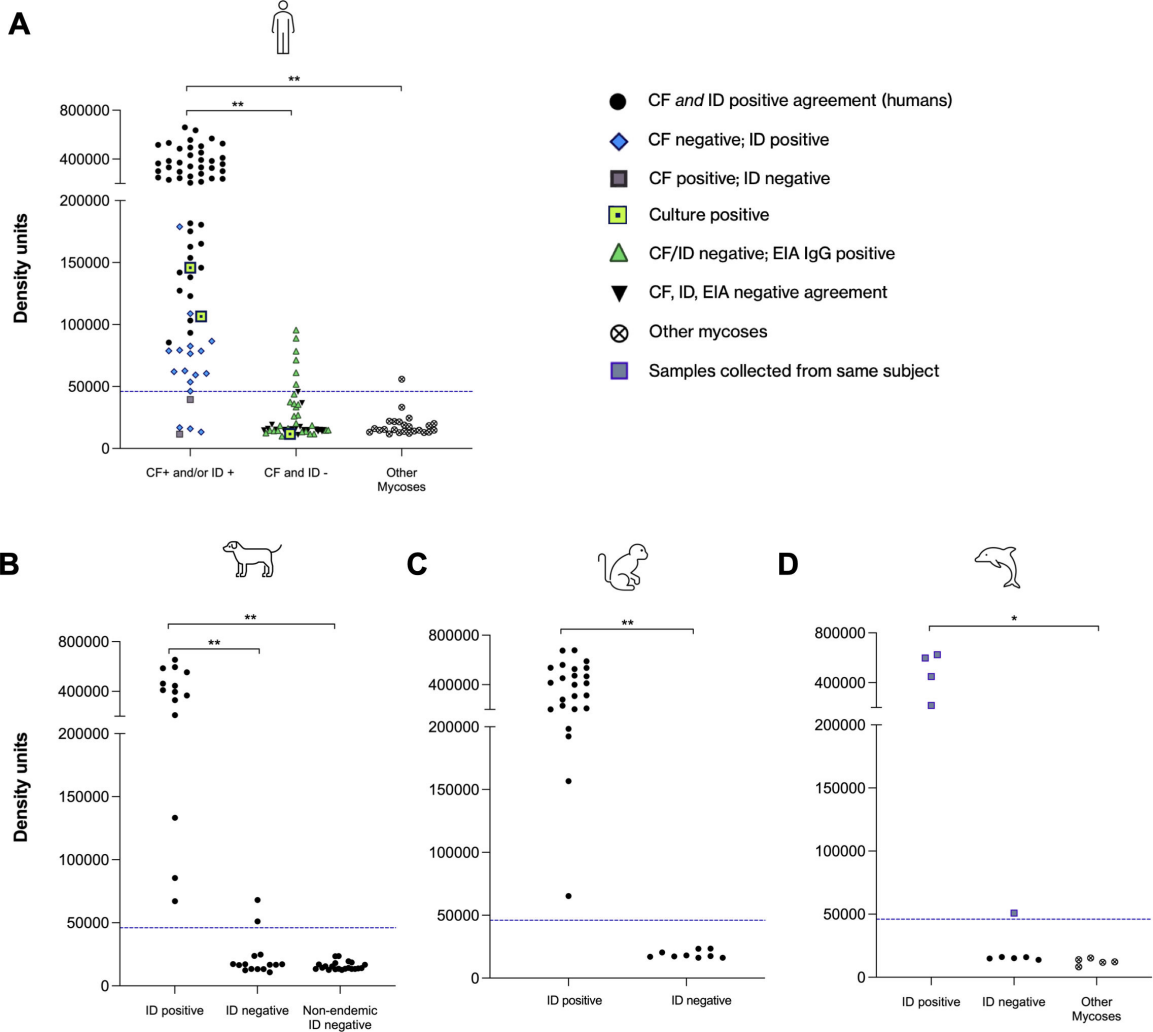
(A) Density units for 143 human serum samples tested by CTS1 LFA. The group on the left of the graph includes patients who were positive by CF and/or ID: samples that were positive by both CF and ID are represented by black circles; samples positive by CF but negative by ID (IgG and/or IgM) are shown as grey squares; and samples negative by CF but positive by ID (IgG and/or IgM) are shown as blue diamonds. The group in the middle of the graph includes patients who were negative by CF and ID: samples that were positive by EIA IgG are shown as green triangles and samples negative by EIA are shown as black inverted triangles. The group on the right of the graph shows patients with positive serology to other endemic mycoses represented with an X encompassed in a circle. Three patients tested were *Coccidioides* culture positive and are denoted as yellow squares with a black center. A cutoff for positivity at 46,000 density units is shown as a blue dashed line. (B) Density units for 50 canine serum samples tested, separated by ID results for endemic samples, and nonendemic samples on the right of the graph. (C) Density units for 33 macaque plasma samples tested, separated by ID results. (D) Density units for 15 dolphin serum samples tested, separated by ID results. Purple squares represent longitudinal samples from the same dolphin collected over 5 years. Dolphins with other mycoses are designated with an X encompassed in a circle. Statistical analysis was performed using Wilcoxon rank sum test with Bonferroni correction (*P ≤ 0.01 and **P < 0.001).

A set of 143 randomly selected sera from the 323 samples tested by ELISA were run on the LFA to evaluate its performance. Additionally, serum and plasma from canines, macaques, and dolphins were evaluated by the rapid test. The test line density units obtained for each sample are illustrated in Fig. 3, separated by species. For humans, the performance of the CTS1 LFA and sōna LFA will predictably change based on the reference standard used as a comparator. If we consider a true positive to be any patient who returned a positive result by CF and/or ID, regardless of agreement, a cutoff of 46,000 test line density units results in detection of 92.7% of human sera, missing only five specimens. We applied the same test line cutoff value (46,000) calculated for human samples to serum and plasma samples from canines, macaques, and dolphins which yielded similar performance (Fig. 3B through D). Significant differences between groups are shown in Fig. 3. Precision testing was performed on replicate samples (*n* = 10) and showed a CV of ~14% from an antibody-negative serum sample and ~11% in an antibody-positive serum sample.

### Correlation of CF and quantitative ID titers with LFA density units

**Fig 4:**
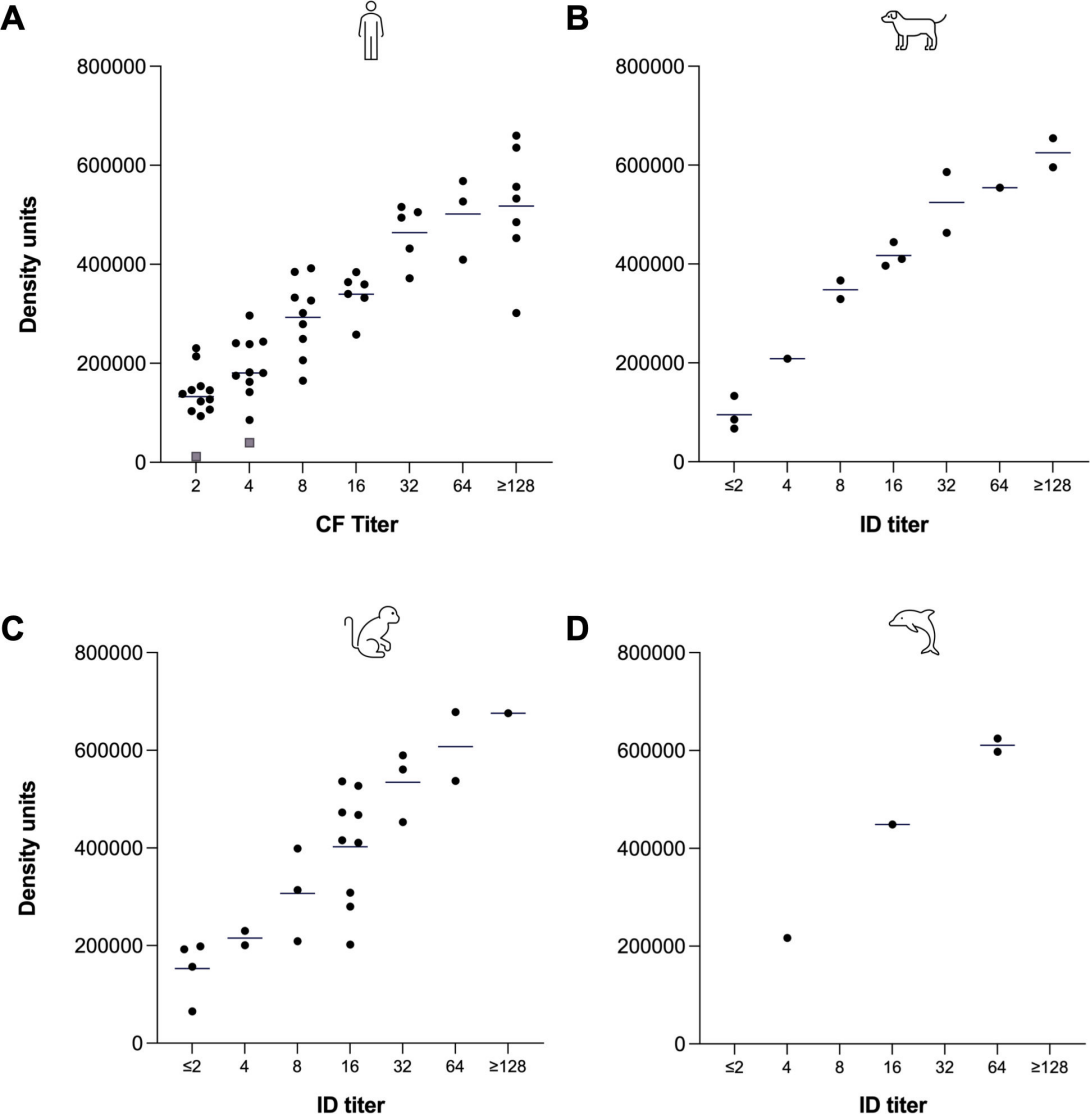
Observed relationship between antibody titer by CF or ID and LFA density units in (A) human, (B) canine, (C) macaque, and (D) dolphin samples. For each titer group, a line represents mean density units. Gray squares in (A) represent samples that were positive by CF but negative by ID.

Being able to quantitate antibody levels detected by the LFA as density units provided an opportunity to determine if there was any correlation with antibody titers measured by CF or QID. This is important because antibody titers are used in the veterinary setting and by human healthcare providers to guide treatment decisions (46 - 49). Of all the samples tested, 53 human, 14 canine, 24 macaque, and four dolphin samples had a quantifiable antibody titer by CF or QID. Test line density units obtained for these samples in relation to titer is shown in Fig. 4 with an overall positive correlation observed for each species. In human samples, the LFA test line densities and CF titer showed a significant correlation with each other (*r*_*s*_ = 0.8988, *P* < 0.0001).

### Comparison between CTS1 LFA and sōna LFA

**Fig 5:**
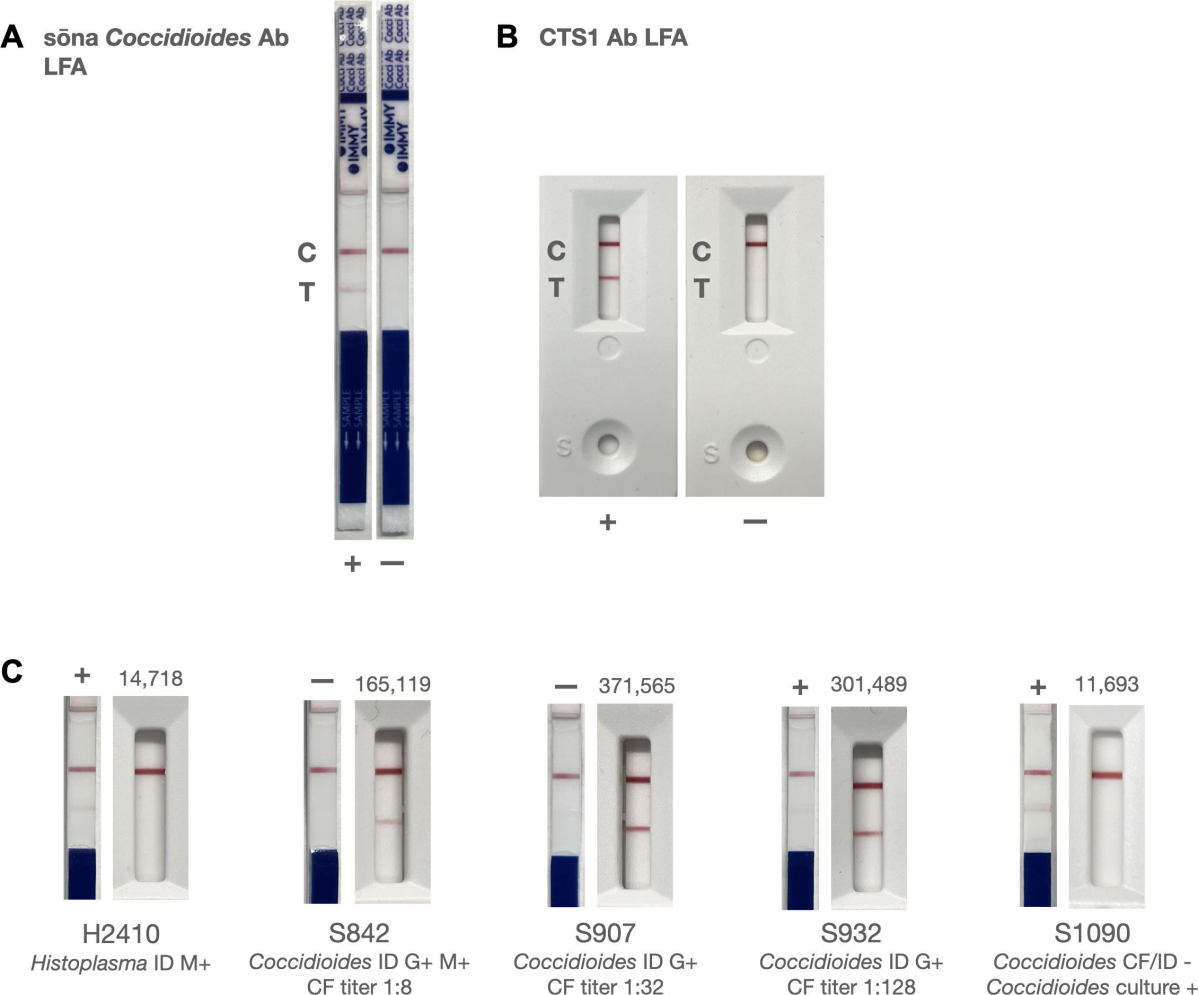
Visual comparison of sōna *Coccidioides* Ab LFA and CTS1 Ab LFA. (A) Positive (+) and negative (−) test results using controls included in the sōna *Coccidioides* Ab LFA kit. (B) Positive (+) and negative (−) test results using VF-positive and VF-negative patient serum, respectively, in the CTS1 Ab LFA. For each test in (A) and (B), control lines are denoted with a “C” and test lines are denoted with a ”T”. (C) Examples of different results produced by the sōna LFA and CTS1 LFA for four patients. Sample H2410 is a nonendemic sample recorded to have positive *Histoplasma* and negative *Coccidioides* serology. H2410 produced a positive result by the sōna LFA and a negative result by the CTS1 LFA (14,718 density units). Samples S842 (CF titer of 1:8) and S907 (CF titer of 1:32) both produced a negative result by the sōna LFA and a positive result by the CTS1 LFA (165,119 and 371,565 density units, respectively). Sample S932 (CF titer of 1:128) produced a positive result by both assays, with a darker line shown by the CTS1 LFA (301,489 density units). Sample S1090 (CF titer negative, *Coccidioides* culture-positive) produced a positive result by the sōna LFA and a negative result by the CTS1 LFA (11,693 density units). Density units for the CTS1 LFA are shown above each test. Diagnostic results by *Coccidioides* ID and CF are shown below each test.

The next comparison of interest was against the only commercially available LFA for coccidioidomycosis. The same 143 human serum samples as above were tested using sōna LFA strips followed by a visual interpretation by two independent individuals. Both LFAs were evaluated for their PPA and NPA using three criteria as follows: (i) when both CF and ID samples were positive; (ii) when either CF or ID results were positive; and (iii) against any single positive serological assay result (EIA IgG and/or ID and/or CF positive). While the number of potential positive samples varied based on the criteria, the negative sample set was consistent and defined as negative by all three methods and/or positive for a different mycosis. By criteria (i), the CTS1 LFA detected all 51 positive samples, while sōna detected only 36/51 (71%) patients (Table 1). As CF and ID criteria for a positive result became less stringent (i.e., more positive samples) in methods (ii) and (iii), the PPA of both assays decreased (Table 1). Because the samples tested by the LFA were randomly chosen, it is difficult to know the expected prevalence of VF infection in the population used; we therefore assumed the prevalence of VF was between 15% and 30% as reported for patients with CAP in Arizona (17, 18) and computed the PPV and NPV of both assays using Bayes’ rule (50) (Table 1). Visual representations of both tests are illustrated in Fig. 5.

**TABLE 1:** CTS1 LFA and sōna LFA performances and 95% CIs compared to other serologic assays expressed as percentages (%)^*a*^

					Prevalence = 15%		Prevalence = 30%	
Assay	Criteria	Total *n* (pos, neg)	PPA % (95% CI)	NPA % (95% CI)	PPV % (95% CI)	NPV % (95% CI)	PPV % (95% CI)	NPV % (95% CI)
CTS1 LFA	(1) CF and ID positive agreement	94 (51, 43)	100 (93.0–100.0)	97.7 (87.9–99.6)	77 (47.3–89.2)	99.5 (97.4–99.8)	89 (68.6–95.3)	98.9 (94–99.6)
	(2) CF and/or ID positive	113 (70, 43)	92.9 (84.3–96.9)	97.7 (87.9–99.6)	87.6 (50.4–98)	98.7 (97.1–99.4)	94.5 (71.1–99.2)	97 (93.2–98.7)
	(3) Anyone positive; CF and/or ID and/or EIA positive	143 (100,43)	71.0 (61.5–79.0)	97.7 (87.9–99.6)	84.3 (43.6–97.4)	95 (93.3–96.3)	92.9 (65.3–98.9)	88.7 (85.2–91.5)
Sōna LFA	(1) CF and ID positive agreement	94 (51, 43)	70.6 (57.0–81.3)	79.1 (64.8–88.6)	37.3 (24.5–52.2)	93.8 (90.6–96)	59.1 (44.1–72.6)	86.3 (80–90.8)
	(2) CF and/or ID positive	113 (70, 43)	64.3 (52.6–74.5)	79.1 (64.8–88.6)	35.1 (22.8–49.9)	92.6 (89.8–94.7)	56.8 (41.8–70.7)	83.8 (78.5–88)
	(3) Anyone positive; CF and/or ID and/or EIA positive	143 (100,43)	51.0 (41.3–60.6)	79.1 (64.8–88.6)	30.1 (18.9–44.2)	90.1 (87.7–92.2)	51.1 (36.2–65.8)	79 (74.5–82.9)

^*a*^ For the CTS1 LFA, a cutoff of 46,000 units differentiated positive and negative samples. Three different criteria were used to evaluate PPA, NPA, PPV, and NPV of each assay.

## DISCUSSION

Here, the development and evaluation of a rapid test that measures levels of anti-CTS1 antibodies are presented. The highlight of this LFA is the speed at which a result can be obtained (10 minutes) compared to that of ID and CF (minimum 48 hours), while maintaining near-equivalent performance. In a first evaluation reported here, the LFA out-performs another commercially available rapid test, sōna, with a faster time to result (10 minutes versus 30 minutes) and better PPA and NPA (Table 1). When our CTS1 LFA is compared to diagnostic results obtained from ID and CF tests used in the clinical laboratory, regardless of agreement between ID and CF results, the CTS1 LFA has 92.9% PPA and 97.7% NPA (Table 1). These agreements are higher than the sōna LFA with 64.3% PPA and 79.1% NPA. The PPAs reported in Table 1 for sōna are in between assay sensitivities reported elsewhere (38, 39). Although the 30–60-minute time-to-result from sōna is an improvement compared to other Food and Drug Administration (FDA)-approved VF diagnostic assays, a 30-minute wait may not be fast enough for emergency departments, urgent care, or primary care visits, where the average visit time does not normally exceed 20 minutes (51). Thus, the CTS1 LFA reported here may allow for clinical decisions to be made in consultation with the patient during a healthcare visit.

In our experience, the sōna LFA is more laborious to set up than the CTS1 LFA. Sōna requires a multistep dilution of serum to a 1:441 dilution, which is subsequently transferred to a flat-bottom tube or plate before addition of the test strip. The CTS1 LFA does not require any dilution of serum, instead adding 6.8 µL of serum or plasma directly to the cassette containing the test strip followed by 60 µL of chase buffer. The CTS1 LFA can then be read by an LFA test reader that provides numerical test line density units. In contrast, the sōna LFA is read visually and is therefore subject to interpretation by the person reading the test. For example, in one study that investigated the use of the sōna LFA with cerebrospinal fluid (CSF), nearly 10% of results was excluded because there was disagreement of the result reported by observers (negative versus weak positive) (52). Although this was investigational as CSF was not FDA-approved for use with sōna at the time, our group had a similar experience with serum where some lines were reported differently by each observer. Utilizing a density reader for the CTS1 LFA eliminated observer interpretation, instead quantitatively measuring test line densities.

The quantitative output of the CTS1 LFA enabled us to determine that test line density units correlated with CF and QID antibody titers (Fig. 4). Positive correlation between numerical test line density units and laboratory-determined titers suggests that test line density units could eventually be used to both help diagnose patients qualitatively and determine antibody titers for ongoing care. Although antibody titers are the currently accepted measurement for longitudinal monitoring of patients with chronic disease, numerical test line density units provided by the LFA reader may allow for more precise monitoring of patients who visit the clinic quarterly, as compared to titers determined by traditional methods. Furthermore evaluation will need to be done to examine this utility.

Another feature of the CTS1 LFA is its use of a single antigen, rCTS1, in contrast to EIA, ID, and CF assays which utilize antigen preparations containing multiple proteins. A distinct advantage of using rCTS1 as a single antigen is that we know the exact concentration and characteristics of rCTS1 in the LFA. This increases precision of the LFA and reduces variability observed with antigen preparations from fungal lysates prepared in BSL3 laboratories, as each preparation may differ between batches. Although one study retrospectively examined the trend of CF antibody titers over the course of antifungal treatment (33), the relationship between levels of antibodies solely against CTS1 and disease progression is not currently known. However, CTS1 is a significant seroreactive protein in CF and ID antigen preparations (29, 43) used for monitoring patient antibody titers longitudinally, which may suggest the observed CF titer dynamics are similarly applicable to CTS1 serologic kinetics.

While using a single coccidioidal antigen (CTS1) from *Coccidioides posadasii* as an antibody target may be a limitation, sequence alignments from six different laboratory and clinical isolates of *C. posadasii* and *Coccidioides immitis* showed no lower than 95% sequence identity at the protein level. Additionally, 93% of sera with positive CF/ID serology that we tested by ELISA contained antibodies that bound CTS1 (Fig. 2). One explanation for the 7% of patients who did not bind CTS1 by our ELISA is that there may be sufficient levels of patient antibodies targeting other coccidioidal proteins such that the ID and/or CF methods were able to produce a positive result. Longitudinal monitoring of serum from patients who either react weakly or not at all with CTS1 may provide some insight on anti-CTS1 antibody kinetics. Furthermore study is needed to investigate this, along with inclusion of specimens from regions of *C. immitis* endemicity.

Limitations of antibody-based detection of VF include the possibility of falsely negative results for immunocompromised patients (53 - 55) and patients with delayed antibody response despite signs of clinical illness (56, 57). Additionally, antibodies may be detectable at low titers for months to years after disease resolution (22). This underscores the shortcomings of antibody-based assays, including the one presented here, as a host response instead of a component of the organism itself is being detected. In this study, three patients tested by ELISA and LFA were culture positive, two of which were positive by CF and ID, the other negative by CF and ID but positive by EIA. This demonstrates the variability of antibody responses and discordance among antibody-based diagnostic assays. The sōna LFA was able to detect antibodies from all three of these patients, while the CTS1 LFA detected antibodies from the two patients positive by CF and ID. A different group had the opposite experience, whereby sōna failed to detect three culture positive patients who were positive by EIA (38). Although it is extremely important that these patients be diagnosed as soon as possible to avoid inappropriate treatment, both sōna and our CTS1 LFA are antibody based, not antigen based. Still, the failure to detect one culture-positive patient (Fig. 5C, S1090) further highlights the need for a rapid antigen-based test that can detect any component of the fungus, especially in the absence of culture positivity. Alternatively, antigens other than CTS1 could be investigated for their antibody-detection utility.

Despite the weaknesses of antibody-based tests for coccidioidomycosis, rapid point-of-care tests for VF could help healthcare providers make decisions in real-time, usually while the patient is present. In high-prevalence settings, it is vital to rule-in or rule-out a diagnosis of VF quickly so that patients may receive appropriate treatment–or perhaps more importantly, to avoid inappropriate antibacterial therapy. Antibacterial drugs carry risks and improper use contributes to the global crisis of antibacterial resistance. Additional clinical studies are needed to further evaluate the performance of this assay in real-time, as well as longitudinal studies to singularly characterize the CTS1 antibody response over disease course. Our group is currently investigating use of the LFA with cerebrospinal fluid, as well as addition of a whole blood filter to be able to perform the LFA with a finger-stick drop of blood. If this assay can perform in clinical settings similar to how it performed in our research setting, it has the potential to allow for rapid monitoring of patients with chronic VF infection, such that a healthcare provider could know the patient’s titer during the visit and discuss treatment options with the patient. This is also true for veterinary settings, where rapid monitoring of the antibody status in VF-suspect or VF-confirmed cases can aid in real-time decision-making for either initiating or adjusting treatment while the pet owner and veterinarian are in the same room.

## Data Availability

The raw data of this study are available from the corresponding author, D.F.L., upon reasonable request.
